# Activity of the acyl-CoA synthetase ACSL6 isoforms: role of the fatty acid Gate-domains

**DOI:** 10.1186/1471-2091-11-18

**Published:** 2010-04-29

**Authors:** Eric Soupene, Nghi Phuong Dinh, Melvin Siliakus, Frans A Kuypers

**Affiliations:** 1 Children's Hospital Oakland Research Institute, Oakland, California 94609, USA

## Abstract

**Background:**

Activation of fatty acids by acyl-CoA synthetase enzymes is required for *de novo *lipid synthesis, fatty acid catabolism, and remodeling of biological membranes. Human long-chain acyl-CoA synthetase member 6, ASCL6, is a form present in the plasma membrane of cells. Splicing events affecting the amino-terminus and alternative motifs near the ATP-binding site generate different isoforms of ACSL6.

**Results:**

Isoforms with different fatty acid Gate-domain motifs have different activity and the form lacking this domain, isoform 3, showed no detectable activity. Enzymes truncated of the first 40 residues generate acyl-CoAs at a faster rate than the full-length protein. The gating residue, which prevents entry of the fatty acid substrate unless one molecule of ATP has already accessed the catalytic site, was identified as a tyrosine for isoform 1 and a phenylalanine for isoform 2 at position 319. All isoforms, with or without a fatty acid Gate-domain, as well as recombinant protein truncated of the N-terminus, can interact to form enzymatic complexes with identical or different isoforms.

**Conclusion:**

The alternative fatty acid Gate-domain motifs are essential determinants for the activity of the human ACSL6 isoforms, which appear to act as homodimeric enzyme as well as in complex with other spliced forms. These findings provide evidence that the diversity of these enzyme species could produce the variety of acyl-CoA synthetase activities that are necessary to generate and repair the hundreds of lipid species present in membranes.

## Background

In mammals, long-chain acyl-CoA synthetases (ACSLs) are necessary for fatty acid degradation, phospholipid remodeling, and the production of long acyl-CoA esters that regulate many physiological processes [1]. These membrane-bound enzymes act on non-polar hydrophobic substrates, fatty acids, generating acyl-CoAs, important activated intermediates in lipid synthesis pathways, that are water-soluble as well as powerful detergents [2-7]. In the first step of the two-step reaction catalyzed by these enzymes, an acyl-AMP intermediate is formed from ATP. AMP is then exchanged with co-enzyme A to produce the activated acyl-CoA. The five long-chain acyl-CoA synthetase genes can give rise to multiple spliced transcript variants [1]. The structure of these membrane proteins has not been solved for the mammalian ACSLs but homology to a bacterial form, whose structure has been determined, points at specific structural features that are important for these enzymes across species [1,2,8-10]. In particular, the bacterial form acts as a dimer, and a conserved short motif, called the fatty acid Gate domain, is implicated in controlling access of the fatty acid substrate to the catalytic site of each of the two sub-units [2].

These membrane proteins can not be purified in the absence of detergent and can form aggregates when over-expressed in *E. coli *[1]. Defining the enzymatic characteristics of the mammalian forms *in vivo *as well as *in vitro *has proven a challenge and has resulted in rather contradictory results [1]. Isoforms of the human ACSL6 members, predominately found in brain tissue and erythrocytes, can be expressed as active enzymes in the membrane of *E. coli *[8,11-13]. We determined the activity of several isoforms representing alternative spliced variants which differ at their amino-terminus and have different short motifs of 26 residues in close proximity of the ATP-binding site [8]. These alternative motifs, specific for the mammalian forms, were predicted to contain different versions of the bacterial homologue fatty acid Gate-domain [2,8,14,15]. We now show that alternative splicing of these motifs generates isoforms of ACSL6 with strikingly different activity, and we identified the aromatic residues representing the Gating residue for fatty acid entry for each version of the Gate-domains of human ACSL6.

## Results and Discussion

### Activity of the Tyrosine-Gate, Phenylalanine-Gate and no-Gate domain isoforms

**Figure 1:**
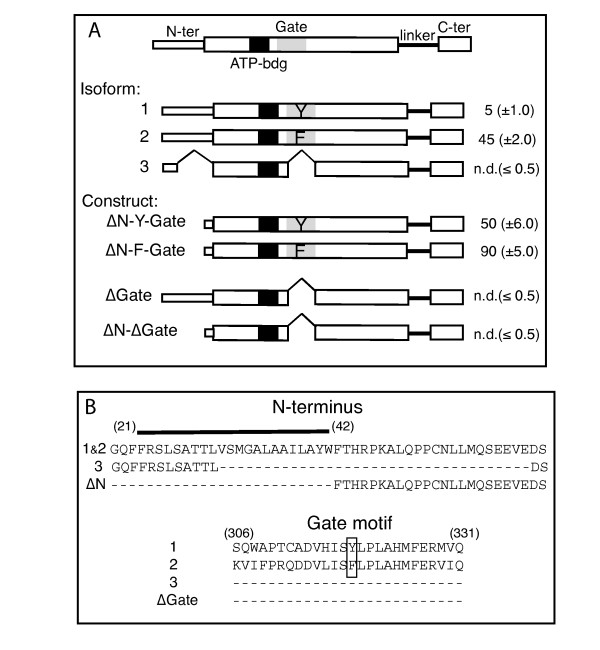
**Human ACSL6 isoforms and truncated constructs**. **A**. Cartoon representation of the three spliced isoforms, and of the four truncated constructs studied in this work. Isoform 1 and 2 represent spliced forms with a Gate-domain containing a tyrosine (Y) or a phenylalanine (F) at position 319, respectively. Isoform 3 has a short internal deletion at the N-terminus, and has no Gate-domain. Numbers on the right of each of the construct represent the acyl-CoA synthetase activity rate values (nmol acyl-CoA formed per mg proteins per min) obtained with oleic acid (6 μM), and the standard deviations of 3 measurements are given in parenthesis. Note that for isoform 3 and the two constructs ΔGate and ΔN-ΔGate (see text), activity values were as low as the values obtained with control cells transformed with the empty vector alone (values of ≈0.5) and could not be reliably detected (not detected, n.d.). **B**. Amino acids alignment of the N-terminus (top) and of the Gate-domain motif (bottom). A predicted trans-membrane spanning segment (amino acid 21 to 42) at the N-terminus is indicated for isoform 1 and 2. The two predicted aromatic Gating residues, Y^319 ^and F^319^, of the Gate-domains are boxed. Note the presence of H^316 ^in isoform 1 and of L^316 ^in isoform 2.

On the basis of sequence similarity with the Tryptophan-containing Gate-domain of the bacterial form, we have proposed that human ACSL6 isoform 1 contains a Tyrosine-containing Gate-domain (referred to as Y-Gate), whereas isoform 2 contains a Phenylalanine-containing Gate-domain (referred to as F-Gate) [1,8]. For isoform 3, the two alternative spliced exons are skipped and this enzyme lacks a Gate-domain (referred to as no-Gate) (Figure 1). Isoform 3 also represents alternative usage of a splicing site affecting the N-terminus resulting in a short truncation (Figure 1). These three isoforms and several recombinant mutant forms were produced in *E. coli*. Although a significant amount of the expressed proteins accumulated in inclusion bodies, mammalian ACSL forms can be expressed in their active form in the plasma membrane of *E. coli *[8,12]. Under the growth conditions used to produce the recombinant forms, the bacterial ACSL form, FadD [9], is expressed at a very low level [9,12]. This presents a unique advantage of the bacterial expression system in contrast to the expression of these isoforms in eukaryotic cells. In those cells, the endogenous ACSL enzymes would interfere with measurement of the activity of the recombinant ACSL6 forms. All forms and mutants used in this study are presented in Figure 1. Few differences in expression level of the isoforms and constructs were observed in the different membrane fractions obtained from *E. coli *(Additional file 1, and not shown).

**Figure 2:**
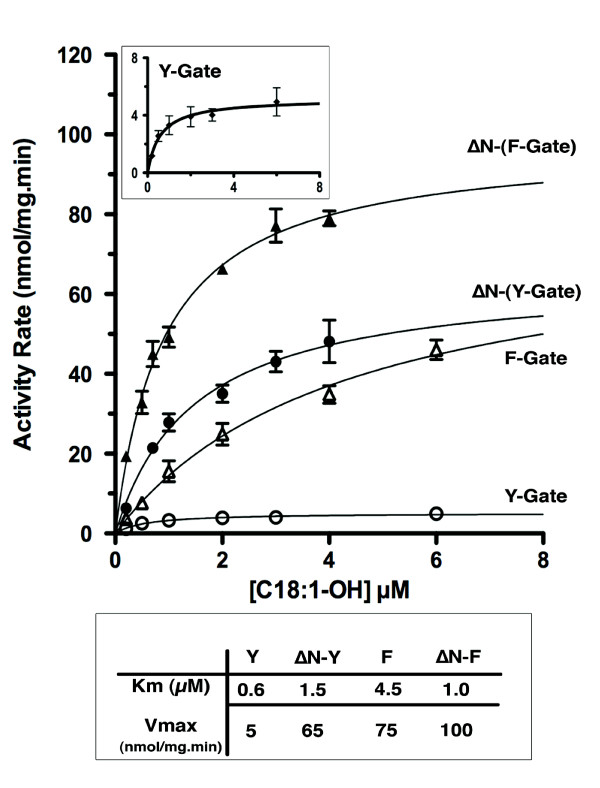
**Acyl-CoA synthetase activity of ACSL6 isoforms**. Activity rate values (nmol of C_18:1_-CoA formed/mg of membrane proteins per min) of isoform 1 (Y-Gate), isoform 2 (F-Gate) and their respective N-terminus truncated forms (ΔN-Y-Gate, ΔN-F-Gate) are represented as function of the concentration of the substrate oleic acid. For each concentration of (^14^C)-C_18:1_-OH, rates were obtained by measurements at 30°C of the formation of C_18:1_-CoA at 1, 2 and 3 minutes after initiation of the reaction with 3 μg of membrane proteins for isoform 1, 1 μg for isoform 2 and 0.25 μg for the 2 ΔN-truncated forms. Each value represents the average of 4 different measurements and the standard deviation is indicated as error bars. ***Inset top***: Activity of isoform 1 was low and is shown with a different y axis. ***Inset bottom***: Kinetic parameters [K_*m *_(μM) and Vmax (nmol/mg.min)] were calculated from these data using Prism 5.0 program (see Methods) and are shown in the table.

The three versions of the human protein, Y-Gate (isoform 1), F-Gate (isoform 2) and no-Gate (isoform 3), have remarkable different activities (Figure 1 and 2). In presence of oleic acid (C_18:1_-OH), formation of oleoyl-CoA (C_18:1_-CoA) by the F-Gate enzyme was about 10 times higher than that of the Y-Gate enzyme (45 ± 2 vs. 5 ± 1 nmol/mg. min) (Figure 1, right column). The activity of isoform 3, which has no Gate-domain and a short internal deletion at its N-terminus, was so low that it could not reliably be distinguished from the activity values obtained from control membrane fraction isolated from cells that did not express the human enzyme (0.6 ± 0.4 as compared to the detection limit of ≤0.5 nmol/mg. min) (Figure 1). In addition, a recombinant full-length version of an enzyme without a Gate-domain, construct ΔGate, displayed no detectable activity (Figure 1). Reactions performed with other fatty acid substrates, palmitic acid (C_16:0_-OH) and arachidonic acid (C_20:4_-OH), did not result in formation of acyl-CoA products by these two inactive forms and acyl-AMPs intermediate species were also not detected (data not shown and see below). These results established that the fatty acid Gate-domain is essential for the activity of the human ACSL forms.

### Identification of the Gating residues

**Figure 3:**
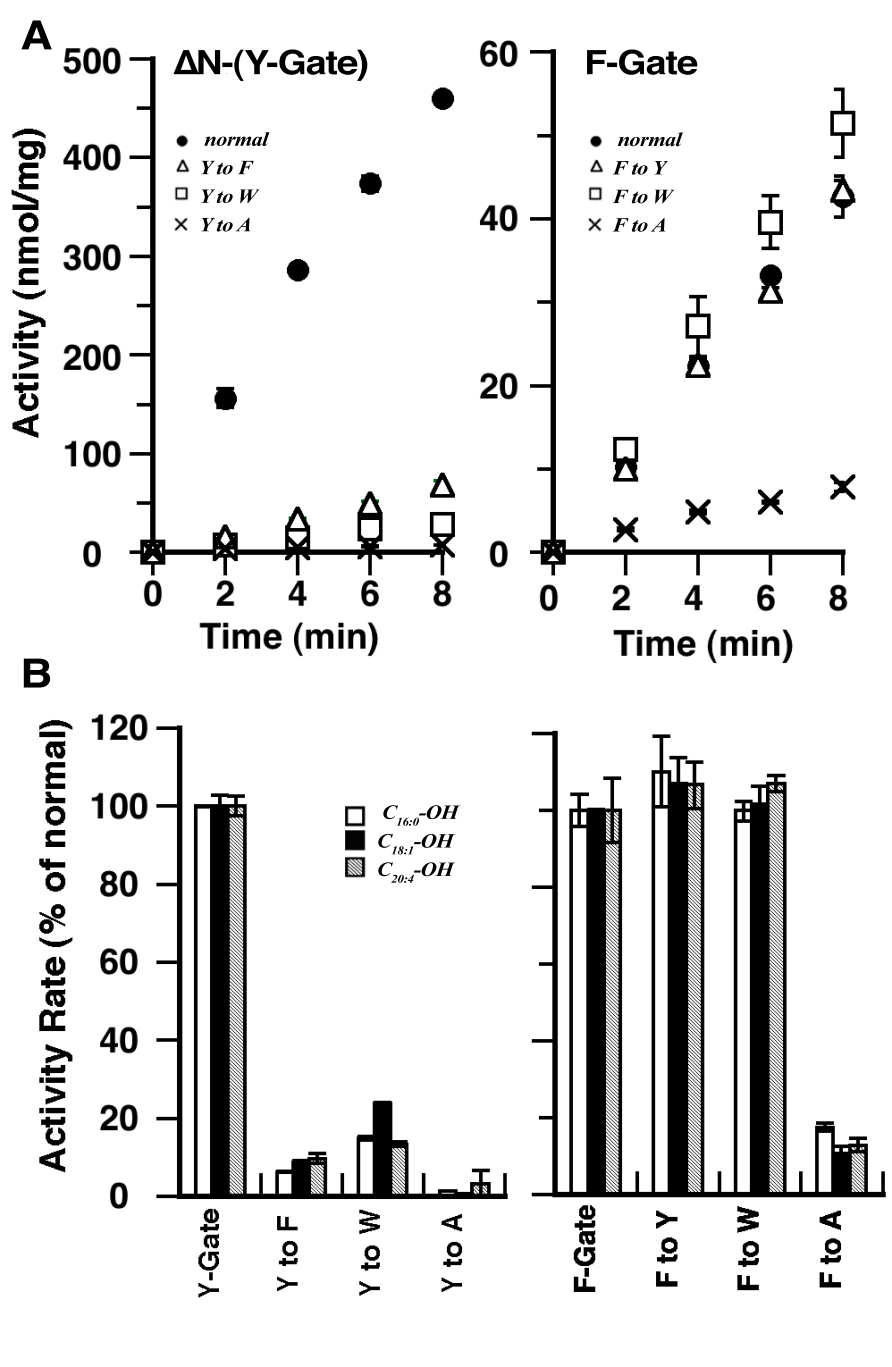
**Role of the aromatic residue at position 319**. The tyrosine residue of isoform 1 (Y-Gate) was changed by site-directed mutagenesis to a phenylalanine (Y to F), a tryptophan (Y to W) and an alanine (Y to A). The phenylalanine residue of isoform 2 (F-Gate) was changed to a tyrosine (F to Y), a tryptophan (F to W) and an alanine (F to A). Because activity of isoform 1 was low, its amino-terminus was removed to increase activity of the mutant forms. Data obtained with ΔN-Y-Gate construct and full-length F-Gate isoform are presented. Measurements were also performed with the full-length Y-Gate version and confirmed findings obtained with the ΔN version. Average and the standard deviation of 3 different experiments are shown. **A**. Activity obtained with the ΔN-Y-Gate mutants set (left) and with the F-Gate mutants set (right) in presence of 5 μM (^14^C)-C_16:0_-OH. Left panel: *normal *Y-Gate (filled circles); Y^319 ^to W^319 ^(triangles); Y^319 ^to F^319 ^(squares); Y^319 ^to A^319 ^(crosses). Right panel: *normal *F-Gate (filled circles); F^319 ^to W^319 ^(triangles); F^319 ^to Y^319 ^(squares); F^319 ^to A^319 ^(crosses). **B**. Activity measurements were performed with 5 μM (^14^C)-C_16:0_-OH (open bar), (^14^C)-C_18:1_-OH (filled bar) or (^14^C)-C_20:4_-OH (hatched bar). Activity rates were calculated from 0 to 8 minutes (as shown in panel A). The values obtained for the *normal *forms were arbitrary set at 100%, and the activity rates of the corresponding mutated Y or F-Gate form, as indicated, were calculated relative to them.

In the bacterial ACSL protein, the aromatic tryptophan residue blocks entry of the fatty acid in the absence of ATP. Upon ATP binding to the nearby P-loop, a conformational change induces rotation of the protruding side-chain ring of tryptophan away from the center of the fatty acid channel allowing entry of the fatty acid substrate [2,8]. This tryptophan gating residue, part of the fatty acid Gate-domain, is not conserved in mammalian ACSL. The two others aromatic residues at position 319, tyrosine or phenylalanine, were predicted to represent the gating residues of the two alternative versions of the human ACSL6 Gate-domain of isoform 1 and 2, respectively [8]. By site-directed mutagenesis, the tyrosine residue of isoform 1 was replaced by phenylanine (Y-to-F mutant), tryptophan (Y-to-W mutant) or alanine (Y-to-A mutant). Similarly, the phenylalanine residue of isoform 2 was changed to a tyrosine (F-to-Y mutant), tryptophan (F-to-W mutant) or alanine (F-to-A mutant). Because the activity of the Y-Gate isoform was very low, these tests were also performed with the more active version obtained by removal of the first 40 residues, ΔN-(Y-Gate) construct (Figure 1, 2 and Additional file 1). Data obtained with this truncated form are presented. Substitution of the aromatic residues, tyrosine and phenylalanine, to an alanine (Y-to-A and F-to-A mutants) produced forms with very low activity (Figure 3). This effect was independent of the fatty acid chain length (C_16_, C_18 _or C_20_) and relative saturation or unsaturation (0, 1 or 4 double bonds) (Figure 3, lower panels). Additional evidence of the essential function of these two aromatic residues was obtained by their substitution by the two other aromatic residues: Y residue of isoform 1 [ΔN-(Y-Gate) construct] replaced by F or W, and F residue of isoform 2 replaced by Y or W. For the ΔN-(Y-Gate) protein, substitution of the tyrosine by a phenylalanine (Y-to-F) or tryptophan (Y-to-W) greatly decreased the activity rate of these mutants (Figure 3, lower left panel). However, in the context of a F-Gate domain, all three aromatic residues (F, Y, W) resulted in enzymes with high activity rates (Figure 3, lower right panel).

These results indicate that the splicing event affecting the two alternative fatty acid Gate-domains of the human and rodent ACSL6 forms produces enzyme species with different Gating residues and enzymatic properties. In addition, these results establish that, for a Y-Gate enzyme, the presence of a tyrosine residue at position 319 was essential for activity, whereas any aromatic residue could be tolerated in the F-Gate domain. Finally, the difference of activity of the Y-to-F mutant compared to a *normal *F-Gate enzyme, and of a F-to-Y mutant compared a *normal *Y-Gate enzyme indicate that other residues are involved in defining the specific activity of isoform 1 and 2.

### Specific Gate-domain residues essential for activity

**Figure 4:**
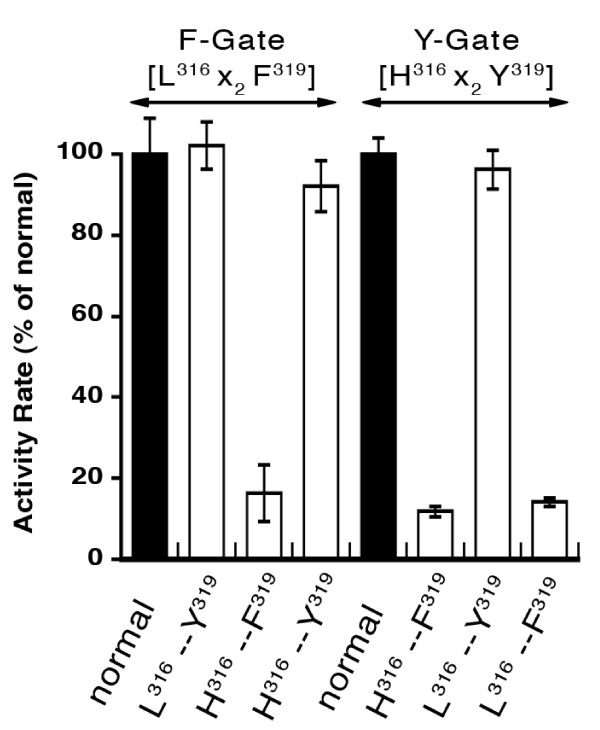
**Residues essential for activity of the two fatty acid Gate-domains**. Leucine 316 of isoform 2 (F-Gate) was changed to a histidine in the *normal *F-Gate form to generate the mutant H^316 ^- F^319 ^and in the mutant (F-to-Y)^319 ^to generate the double mutant H^316 ^- Y^319^. Similarly, histidine 316 of isoform 1 (Y-Gate) was changed to a leucine in the *normal *Y-Gate form to generate the mutant L^316 ^- Y^319^, and in the mutant (Y to F)^319 ^to generate the double mutant L^316 ^- F^319^. Activity measurements were performed with 5 μM (^14^C)-C_18:1_-OH as described in legend of **Figure 2**. Activity rate of the *normal *forms (either the F-Gate or the Y-Gate; filled bars) was set at 100% and values obtained with the corresponding mutated forms were calculated relative to these. Data obtained with ΔN-Y-Gate construct and full-length F-Gate isoform are presented (see legend **Figure 3**). Average and the standard deviation of 3 different experiments are shown.

Among the 26 residues constituting the two alternative spliced Gate-domains, the gating residue and 10 additional residues are different. Thus, a mutant form with a Y to F change still contains 10 residues present in the Y-Gate form that are absent in the *normal *F-Gate form. In the absence of sufficient structural knowledge on these domains, we compared the amino acid sequence of the mammalian, plants and bacterial forms [8] and sought residues only found, or never found, in combination with either the tyrosine or the phenylalanine 319. Using this approach [8], we identified a histidine/leucine residue pair at position 316 that seemed associated with the tyrosine/phenylalanine pair at 319 (Figure 1 and 4). As presented below, whenever a histidine was present at position 316, enzymes with a tyrosine at position 319 were active, whereas the presence of a histidine was sufficient to inactivate a form with a phenylalanine. Histidine 316 is present in the Y-Gate isoform of ACSL6 and ACSL1 (the only other ACSL member that can generate the two versions of the Gate-domain [8]). Histidine 316 is replaced by a leucine in the F-Gate version (Figure 1) of both ACSL6 and ACSL1 [8]. As shown in Figure 4, when a histidine was introduced in place of leucine 316 of the F-Gate isoform (L_normal_^316 ^- F_normal_^319 ^mutated to H_mutant_^316 ^- F_normal_^319^; 3^rd ^bar of graph), the mutant form had an activity nearly as low as the ΔN-(Y to F)^319 ^mutant in the Y-Gate environment, i.e. H_normal_^316 ^- F_mutant_^319^, 6^th ^bar. Substituting the phenylalanine of the poorly active mutant, H_mutant_^316 ^- F_normal_^319 ^(3^rd ^bar), to a tyrosine, generated the double mutant enzyme H_mutant_^316 ^- Y_mutant_^319 ^in the context of a F-Gate motif (4^th ^bar) resembling a *normal *Y-Gate domain (H_normal_^316 ^- Y_normal_^319^, 5^th ^bar), fully restored activity. Hence, a histidine residue at position 316 cannot be present when a phenylalanine residue is also present at position 319 (3^rd ^and 6^th ^bars). However, a Gate-motif with a tyrosine (*natural *or introduced) can accommodate both a leucine and a histidine (2^nd^, 4^th^, 5^th ^and 7^th ^bars). Whereas enzymes with a F-Gate domain can accommodate a tyrosine in place of the phenylalanine regardless of the residue present at position 316 (2^nd ^and 4^th ^bars), enzymes with a Y-Gate motif were only active when a tyrosine was present at position 319 (5^th ^and 7^th ^bars versus 6^th ^and 8^th ^bar). These results are a strong indication that other essential residue(s) may be necessary to permit correct folding and/or function of this domain of the enzyme. In addition to the histidine/leucine pair at position 316, other residues, not necessarily specific to a Gate motif nor necessarily present in the Gate-domain, are involved in defining the specific activity of the isoforms.

### ATP requirement and inhibition of activity

**Figure 5:**
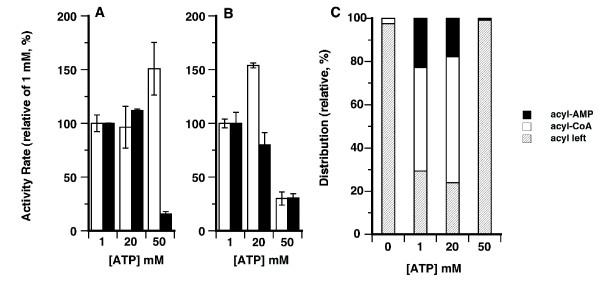
**ATP requirement and detection of acyl-AMP intermediate species**. **A **and **B**. Activity measurements were performed as described in the legend of **Figure 2 **with the full-length isoforms (open bar) and the respective ΔN-truncated versions (filled bar) of isoform 1 (Y-Gate) (panel A) and of isoform 2 (F-Gate) (panel B). Assays were performed in presence of 10 μM (^14^C)-C_18:1_-OH and ATP concentrations were 1, 20 and 50 mM, as indicated. Relative activity rate values are presented in percent of the rate values obtained in presence of 1 mM of ATP. The actual differences in activity of these enzymes are shown in **Figure 2**. For each ATP concentration value, the average and the standard deviation of 3 to 5 different experiments is shown. **C**. Distribution of (^14^C)-C_18:1_-OH (acyl leftover), (^14^C)-C_18:1_-AMP (acyl-AMP) and of (^14^C)-C_18:1_-CoA (acyl-CoA) present in reactions performed without (0), or with addition of 1, 20 and 50 mM ATP, as indicated. Reactions were performed with 10 μM (^14^C)-C_18:1_-OH and 1 μg of isoform 2 for 10 minutes at 30°C. Compounds were spotted on silica plates and separated by thin-layer chromatography. For each radiolabeled compound, the intensity of the spot was quantified, and its contribution to the total radiolabeled intensity under each condition was calculated as follows: (^14^C-species/sum of all ^14^C -species)*100. Note that at 50 mM ATP, the amount of unreacted (^14^C)-C_18:1_-OH (acyl leftover) was as high as under condition without addition of ATP (0 mM).

As indicated above, isoform 2 carrying an F-Gate domain was more active than the Y-Gate isoform. The two rat forms ACSL6_v1 (Y-Gate) and ACSL6_v2 (F-Gate) (≥90% amino acid identity to the human forms) were also reported to have a different activity toward oleic acid [14]. The rat Y-Gate form had a lower activity than the F-Gate form, confirming the results obtained with the human forms, but they also appeared to have a significant difference of affinity for ATP. Rat ACSL6_v1 (Y-Gate) was reported to have a remarkable poor affinity for ATP, K_m _= 12, 210 μM [14], as compared to an already low affinity (K_m _= 1, 480 μM) for the F-Gate form [14]. In our system, the human forms did not show such high ATP requirements. Activity rates did not differ significantly at ATP concentrations of 1, 5 and 20 mM (Figure 5, panel A and B). Figure 5 shows activity rate values obtained with the different ATP concentrations relative to value obtained with 1 mM ATP. Higher concentrations of ATP resulted in a small increase of activity for the form with a low activity (Y-Gate) but these concentrations were strongly inhibitory for the more active F-Gate form and the two very active N-terminus truncated forms (ΔN-constructs, see below). Given that inhibition at high ATP concentration was seen with forms carrying the two versions of the Gate-domain (F, ΔN-Y and ΔN-F), it seems unlikely that the lack of inhibition of the lesser active form, Y-Gate, represents a significant enzymatic difference toward ATP of the two versions of the Gate-domain, as previously suggested for the rat ACSL6 isoforms [14].

ATP is needed for the initial formation of the acyl-AMP intermediate in the two-step reaction catalyzed by ACSL. CoA-SH is subsequently exchanged to form the final acyl-CoA product. To determine if excess ATP could inhibit the CoA-exchange reaction and limit the formation of the product by the active enzymes, all ^14^C-labeled compounds present in the reaction mixture were separated, identified and quantified by thin-layer chromatography (see Methods section). The distribution of the ^14^C-labeled compounds was similar at 1 and 20 mM ATP. Under these conditions, 70-80% of the substrate was converted and the relative ratio of the two acyl-AMP and acyl-CoA species was similar, despite a 20-fold difference in ATP concentration (Figure 5, panel C). However, for reactions performed at 50 mM ATP, the acyl-AMP and acyl-CoA species were not formed, and most of the ^14^C-label was found in the unreacted acyl substrate (acyl left), as was the case for the reaction performed without addition of ATP (0 mM column). Hence, the inhibitory effect of ATP was also observed on the formation of the intermediary acyl-AMP species and accounted for the decrease in production of the final acyl-CoA product. As this effect was not observed with an enzyme lacking strong activity, these results may indicate that the released pyrophosphate at the first-step and/or the released AMP during the second step can inhibit the acyl-AMP reaction. The reported higher K_m _value for the lesser active rat ACSL6 isoform 1 may have been erroneously determined because of the lack of inhibition and of the slightly increased activity obtained at high ATP concentration, as observed with human isoform 1.

### Inhibition of acyl-CoA formation by Triton X-100

**Figure 6:**
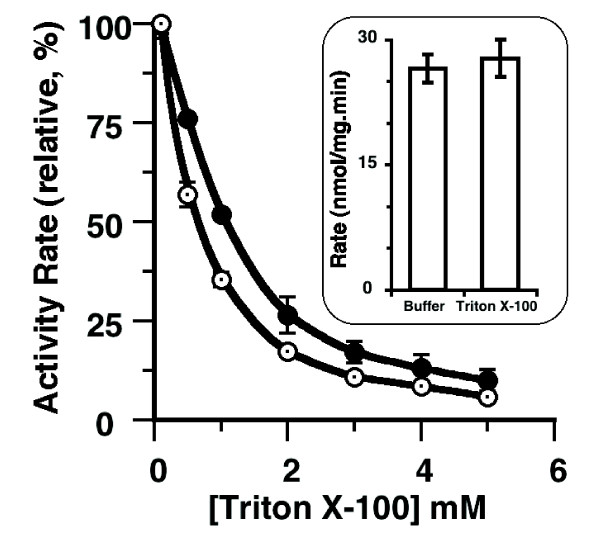
**Effect of Triton X-100 on activity**. Activity measurements were performed with 5 μM (^14^C)-C_18:1_-OH as described in legend of **Figure 2**, with isoform 2 (F-Gate) (filled circles) and its N-terminus truncated version, ΔN-(F-Gate) (open circles), in presence of 0.1, 0.5, 1, 2, 3, 4 and 5 mM Triton X-100. Average and the standard deviation of 3 different experiments performed with each concentration are shown. ***Inset***: Isoform 2 was incubated 30 min on ice with either buffer or 15.9 mM (1%) Triton X-100, as indicated. The treated enzyme preparation was then diluted in the reactional mixture to lower the concentration of Triton X-100 to a non-inhibitory concentration of 130 μM. To the reference sample incubated with buffer, Triton X-100 was added to the reaction at a concentration of 130 μM. Average and the standard deviation of 3 different experiments for each condition is shown.

The detergent Triton X-100 has been used to solubilize rodent ACSLs from microsomal membranes at concentrations of 16 mM or above. These values far exceed the critical micellar concentration (CMC≈0.3 mM) and are therefore expected to produce an assay mixture containing a significant amount of mixed micellar structures. It was reported that the activity of different rodent ACSL forms were either inhibited (ACSL4) [14], stimulated (ACSL5) [16], or unaffected (ACSL6 isoform 1 and 2) [14] by Triton X-100 at concentrations above the CMC value. In our assay system, ACSL6 proteins were not solubilized from the membrane and only a low amount of Triton X-100 (70 μM) was added to solubilize the fatty acid substrate. In contrast to the reported results of the rodent ACSL6, which seemed unaffected by this detergent [14], the human ACSL6 isoforms [such as the moderately active isoform 2 and the very active truncated ΔN construct (see below)] were strongly inhibited with an estimated IC_50 _of 1 mM (Figure 6). To explore this apparent difference between the human and rodent form, we treated human isoform 2 with Triton X-100 at an inhibitory concentration of 16 mM, followed by assaying its activity in the presence of a non-inhibitory concentration of 0.13 mM. This treatment did not result in decreased activity (Figure 6, inset), indicating that the detergent does not denature the protein, at least not irreversibly, but can decrease the rate of catalysis of the human ACSL6 enzymes. This concentration dependent inhibitory effect of Triton X-100 on ACSL activity could be the result of micellar structures that are formed above 0.3 mM. These structures most likely attract the hydrophobic fatty acid substrate and as such, may compete with the enzyme, resulting in decreased availability of the substrate. This effect on ACSL6 activity by Triton X-100 suggests that the conclusions for the different ACSL forms should be interpreted cautiously and raises concerns for the usage of this detergent.

### Alteration of the Carboxy-terminal domain affects activity

**Figure 7:**
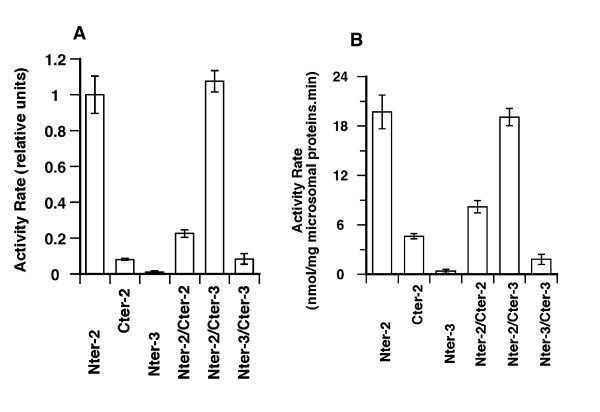
**Activity of differentially tagged forms in single and dual expression system**. Isoform 2 and 3 were cloned in a dual expression vector, which allows production of 1 or 2 proteins tagged with unique epitope either at their N-terminus (N_ter_-isoform) or C-terminus (C_ter_-isoform). Shown are the activity measurements performed with membrane fractions isolated from cells expressing either a single copy of isoform 2 produced with a tag at the N-terminus (N_ter_-2 sample) or at the C-terminus (C_ter_-2 sample), one copy of isoform 3 (N_ter_-3), two copies of isoform 2 (N_ter_-2/C_ter_-2 sample), two copies of isoform 3 (N_ter_-3/C_ter_-3 sample), or one copy of isoform 2 and one copy of isoform 3 (N_ter_-2/C_ter_-3 sample). Measurements were performed with 5 μM (^14^C)-C_18:1_-OH. **A**. Activity rates were normalized to the amount of N-terminally fused proteins present in the different membrane fraction. For the C_ter_-2 sample, this value was calculated relative to the amount of the ACSL6 enzyme present. The normalized values obtained with the sample producing a single protein in its most active version, N_ter_-2 (1^st ^bar), was arbitrary set at a value of 1 and was used to report the relative values obtained with the other samples. **B**. Shown are the activity rate values relative to the amount of the total proteins present in the different membrane fractions and they represent the raw activity data of the normalized values shown in panel A. Averages and the standard deviations of 3 different experiments are shown.

Unexpectedly, an ACSL6 construct with a tag at its C-terminus had a far lower activity than a construct with a tag fused to its N-terminus. As shown on panel A of Figure 7, the relative activity rate (normalized to the amount of ACSL6 protein, as described in the methods section) was 10-times lower for the C-terminally tagged fusion than for the N-terminally tagged construct. Raw activities data are presented in Figure 7, panel B. Fusion at either the N-terminus or C-terminus failed to improve the activity of isoform 3, supporting previous findings that this enzyme was not active (Figure 1 and 7, and not shown). The reduced activity of human ACSL6 (Figure 7) and of mouse ACSL1 [17,18] suggest that addition of residues to the short C-terminus *hammer *domain impair its movement and positioning relative to the *anvil *central domain, and thus, in the formation of the catalytic site [2]. In studies of rat ACSLs, different forms were produced with a FLAG epitope tag at their C-teminus [14,16,19,20]. Activities of these recombinant FLAG-enzymes were not compared to N-terminally tagged proteins and our findings suggest that the kinetic parameters obtained with these recombinants proteins might not represent those of the native forms.

### Removal of the Amino-terminus stimulates activity

**Figure 8:**
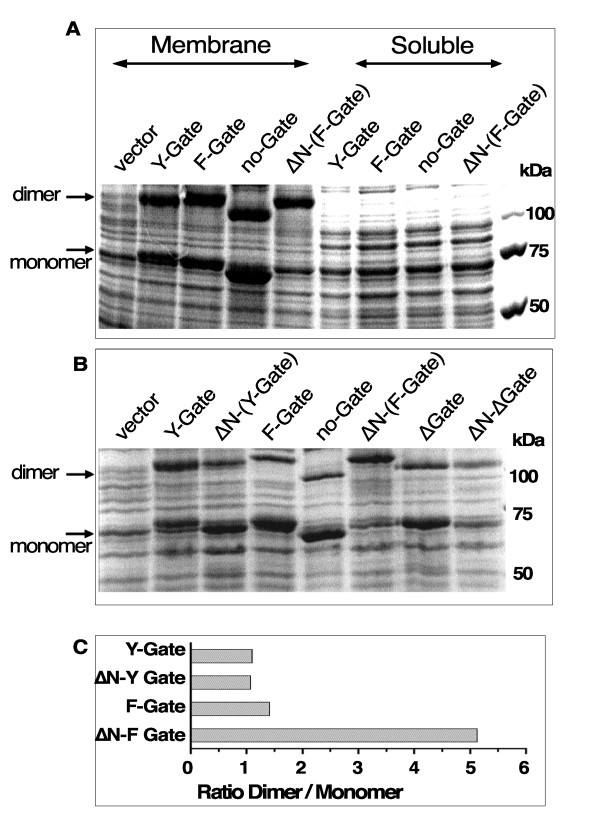
**Detection of isoforms in the membrane of *E. coli***. ACSL6 forms were expressed in *E. coli*. Membrane and soluble fractions were obtained as described in the methods section. Proteins were separated on denaturing SDS-PAGE 7.5% gel and stained with coomassie-blue. The molecular weight standard is shown on the right. Position of the monomer and dimer species is indicated with an arrow. **A**. Proteins present in the membrane (lane 1 to 5) and in the soluble (lane 6 to 9) fractions of *E. coli *carrying the vector and the different isoforms, as indicated, are shown. **B**. Protein present in membrane fractions of *E. coli *carrying the vector, isoform 1 (Y-Gate), ΔN-(Y-Gate), isoform 2 (F-Gate), isoform 3 (no-Gate), ΔN-(F-Gate), ΔGate and, ΔN-ΔGate are shown. **C**. Histogram of the ratios of the intensity value of the slow to the fast migrating bands of isoform 1 and isoform 2 and of their respective N-terminus truncated version (ΔN-Y-Gate and ΔN-F-Gate) is shown. Density of bands with molecular mass of ≈75 kDa (monomer) and ≈140 kDa (dimer) detected on lane 2, 3, 4 and 6 of the gel shown on panel B were quantified using QuantityOne program (Bio-Rad). Intensity values obtained in lane 1 (*E. coli *proteins only) at the same position on the gel, taking into account the slight difference of migration of the different bands, were subtracted to the intensity value of each of the bands of each of the isoforms and constructs.

In mammalian cells, the various members of the ACSL family have been located in membranes of different organelles [1]. In the host *E. coli*, human ACSL6 forms are also associated with the membrane [8,12]. To define region(s) of the protein responsible for this association, we removed a motif at the N-terminus of the protein which was predicted to constitute a trans-membrane (TM) spanning segment [1,8]. This region partially overlaps the truncation that naturally occurs in the spliced variant isoform 3 (Figure 1). Despite this deletion, isoform 3 was found in the membrane (Figure 8, panel A, lane 4). In *E. coli*, recombinant isoform 1, and isoform 2 truncated of this predicted TM (ΔN constructs) were also associated with the membrane (Figure 8, panel A, lane 5; panel B, lane 3 and 6). The ACSL homologue in bacteria, FadD, has been proposed to associate with the membrane through formation of oligomeric complexes partially associated with the lipid bilayer. Other hydrophobic region(s) of human ACSL6 may interact with the bacterial lipids.

The activities of the truncated forms (ΔN constructs) were assayed with oleic acid (C_18:1_-OH) and compared to that of their respective full-length versions (isoform 1 and 2) (Figure 1 and 2). Surprisingly, removal of the N-terminus resulted in enzymes with far greater activity (Figure 2). These differences were most noticeable in the 10-fold increase in velocity value of the otherwise poorly active Y-Gate form. For the more active F-Gate form, removal of the N-terminus resulted in an increase of the apparent affinity rather than in the V_max _value. Similarly, large increases in activity rates were observed with palmitic acid (C_16:0_-OH) and arachidonic acid (C_20:4_-OH) (not shown). As anticipated, truncation of the N-terminus did not rescue the activity of the inactive form truncated of its Gate-domain (ΔGate and ΔN-ΔGate) (Figure 1, right column).

### Amino-terminal domain is not essential for oligomer formation

On denaturing polyacrylamide gels, the migration pattern of the truncated F-Gate form was noticeably different compare to the full-length protein. As reported previously [8], when proteins were solubilized from membranes under mild condition (1% SDS at 37°C) rather than boiled before separation on gels containing 0.1% SDS, human ACSL6 isoforms were detected as two bands with apparent molecular mass of 70 kDa and 140 kDa (Figure 8 and Additional file 1). The calculated mass of a monomeric form was 80 kDa (see legend), but as commonly observed for hydrophobic proteins, human ACSL6 migrates faster than the molecular weight standard of the corresponding mass. The species of higher molecular mass was twice the apparent mass of a monomeric protein and since the two bacterial homologues (FadD and ttACS) are dimeric enzymes [1,2,4], it likely represents the dimeric form of human ACSL6. The same pattern was obtained with rat ACSL6 isoform 1 and isoform 2 expressed in *E. coli *(data not shown). The two bands (70 and 140 kDa) were also recognized by an antibody raised against the epitope tag present at the N-terminus (Additional file 1) and by an affinity-purified antibody raised against human ACSL6 [8]. Both bands were also detected in protein extracts of mammalian cells expressing ACSL6 [8]. Although, these two species were detected with all forms and recombinant constructs, their relative amounts were significantly different for the ΔN-(F-Gate) truncated construct (Figure 8. Panel A, lane 3 vs. lane 5; Panel B, lane 4 vs. 6). As seen on a gel, the ratio of high to low molecular mass species was greater for the truncated ΔN-(F-Gate) protein than for its full-length version (Figure 8, panels B and C). Hence, the N-terminal domain is not essential for formation of the dimeric complexes but its removal increased dimer formation and/or stability in the presence of SDS. Based on the findings that the bacterial forms act as dimers, these complexes probably represent the active species of human ACSL6. Their relative increased in abundance could account for the higher activity observed with the truncated F-Gate construct (Figure 2). The ΔN-(Y-Gate) protein was also more active than its full-length version, but even under the mild denaturing conditions we used, we could not detect a difference in the dimer to monomer ratio. Repeated attempts to resolve and quantify these oligomeric forms under non-denaturing conditions [21,22] or by gel filtration chromatography were not successful. Differences in stability of the two full-length forms (isoform 1 versus isoform 2) might also account for their unexpected differences of activity (Figure 2).

### Detection of interaction between isoforms

**Figure 9:**
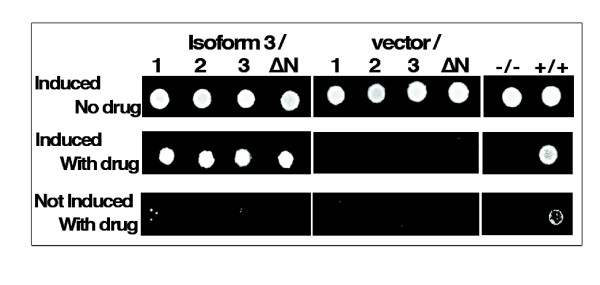
**Two-hybrid interaction analysis of oligomer formation**. Isoform 1, 2 and 3 and ΔN-isoform 2 were cloned in the Bait vector (see Methods). Isoform 3 was cloned in the Target vector. Results obtained with the combination of all isoforms cloned into both vectors as well as the complete set of controls cells and conditions are presented in Additional file 2. Growth tests were performed on solid media in absence (non-selective) or presence (selective) of the histidine biosynthesis inhibitor 3'-AT at a concentration of 5 mM. Expression of the cloned ACSL6 proteins was placed under the control of an IPTG-inducible promotor and tests were performed without (not induced) or with (induced) 30 μM IPTG (see methods). As seen on the control plates and as expected, the *E. coli *cells transformed with empty Bait and Target vectors (-/-) grew in absence of the drug but failed to grow in its presence and, cells transformed with the Bait and Target positive controls (+/+; dimerization domain of Gal4 with interacting domain of Gal11) grew in presence of the drug only when IPTG (induced condition) was present in the medium. Plates on the left side of the figure represent the experimental plates with cells transformed with isoform 3 Target construct and with one of the 4 different Bait constructs, as indicated. Note that, as expected, all cells transformed with the 4 different Bait constructs and with the empty Target vector grew in absence of the drug but not in its presence.

To confirm the presence of dimeric complexes, we monitored their formation using a two-hybrid interaction detection assay. The 3 isoforms and the truncated ΔN version of isoform 2 were fused to the lambda-cI protein, Bait constructs, or fused to the amino-terminal domain of the RNA polymerase alpha subunit, Target constructs (see methods section). In all constructs, fusions were made at the N-terminus of the ACSL6 isoforms and their expression was under the control of an inducible promoter (Additional file 2, panel C and Figure 9). In this system, growth of *E. coli *in the presence of a histidine inhibitor (3'-AT) can only be obtained when the two fusion proteins (Bait and Target) interact to form a dimeric complex (see methods section). Growth was monitored under different conditions and tests were performed either on solid media or in liquid culture, in the absence or presence of the drug, with cells expressing fusion constructs or lacking the constructs. Results of these assays are presented in the Additional file 2 and those obtained on solid media with isoforms 1, 2, 3 and the ΔN construct cloned in the Bait vector in combination with either the empty target vector or with isoform 3 cloned in the target vector are shown in Figure 9. Some combinations of the two vectors with the different isoforms resulted in growth defects even in the absence of the drug (Additional file 2). However, none of the constructs with the inactive isoform 3 used as Target showed a growth defect (Figure 9, top left panel). The cells co-expressing the same isoform (1/1, 2/2 or 3/3) showed growth (Additional file 2), confirming existence of the homodimeric complexes previously detected by electrophoresis (Figure 8, panel A, lane 2, 3 and 4). The ΔN truncated construct rescued growth with all 3 isoforms, providing further evidences that this region is not required for oligomer formation. Heterodimeric interactions were also detected with all forms, especially when the inactive isoform 3 was used as the target (Figure 9, compare left and middle 2^nd ^panels, and Additional file 2). In addition, interactions of the active isoform 1 and 2 with the other forms were detected under both solid and liquid culture conditions (Additional file 2).

### Activity of oligomeric complexes

Addition of a tag at the C-terminus of the active isoform 2 resulted in an enzyme with very low activity. This tagged version of isoform 2 provided an additional test to detect the formation of oligomeric complexes. Activity assays were performed with membrane fractions obtained from cells carrying a single vector that allowed co-expression of two differently tagged proteins from the same promoter. This provided an assay for the measurement of the activity of different isoforms associated in oligomeric complexes. For example, ACSL activity of cells co-expressing each of the two tagged versions of isoform 2, the active N_ter_-2 with the inactive C_ter_-2, were compared to cells that co-express the active N_ter_-2 with the inactive C_ter_-3. In addition, different combinations were made to generate reference and control cells (Figure 7). To detect the putative effects of the inactive sub-unit (C_ter_-2 or C_ter_-3) on activity of the enzyme complex (e.g. N_ter_-2/C_ter_-2 or N_ter_-2/C_ter_-3), the activity rates were normalized to the amount of active sub-units present in the complexes, i.e. N_ter_-2 (see methods section and Figure 7, panel A). When the two different tagged versions of isoform 2 (Figure 7, 4^th ^bar) were co-expressed, the rate of formation of acyl-CoA by membranes obtained from these cells was far lower than the rate of synthesis obtained with the N_ter_-2 enzyme expressed alone (1^st ^bar). Even in the absence of normalization for the relative amount of the active species (Figure 7, panel B), activity rate of the N_ter_-2/C_ter_-2 enzyme was nearly as low as the value obtained with the C_ter_-2 enzyme expressed alone (2^nd ^bar) or with co-expression of the two tagged versions of the inactive isoform 3 (N_ter_-3/C_ter_-3) (Figure 7, last bar). This result strongly suggests that the *inactivated *isoform 2 had a dominant effect on the activity of the active unit, N_ter_-isoform 2. This effect of one protein on the activity of another confirmed that oligomeric complexes were formed as established above. The effect of the inactivated C_ter_-isoform 2 on an active form was compared to the effect of the naturally occurring inactive isoform 3 (the truncated form that misses the essential Gate-domain) on that active form. This test may mimic the *in vivo *circumstances in which various spliced isoforms with different activity rates are expressed in the same cells [1,8]. Intriguingly, interaction with isoform 3 did not result in inhibition of the isoform 2. The species N_ter_-2/C_ter_-3 (Figure 7, 5^th ^bar) was as active as the homodimer N_ter_-2 (1^st ^bar).

The C-terminus *hammer *domain of ACSL is connected to the *anvil *domain, carrying the ATP-binding site and the Gate-domain, by a flexible linker [2]. The *hammer *and *anvil *form the catalytic site and the correct positioning of the C-terminus is essential to prevent escape as well as entry of acyl-AMP until the second step of the reaction with CoA-SH has occurred, and acyl-CoA is formed. The defect of the extended C-terminally tagged enzyme could render the second active sub-unit of the dimeric complex structurally defective resulting in a slower acyl-CoA synthetase activity. Isoform 3 lacks the motif carrying the fatty acid Gate-domain. This domain is essential for the entry and access of the fatty acid to the catalytic site. The removal of the short region connecting the fatty acid tunnel to the ATP-binding site might not preclude processing of fatty acids once they have found their way to the site. The apparent activity of the isoform 3 in the complex formed with isoform 2 could indicate that entry of the fatty acids through one of the sub-units provides substrate to the other one through an internal channel event. Efforts to solve the structure of these homo- and heterodimeric complexes are underway.

## Conclusions

The alternative fatty acid Gate-domain motifs play an essential role in the activity of the human ACSL6 enzymes. The aromatic residues representing the Gating residues of the two versions of the Gate-domain were identified as a tyrosine for isoform 1 and a phenylalanine for isoform 2. A tyrosine and tryptophan could be substituted to the phenylalanine residue of isoform 2 without affecting activity whereas the tyrosine residue was essential for the activity of isoform 1. We also identified another residue, a leucine, present in the F-containing Gate domain (F-Gate), that is essential for the activity of that form. The three isoforms can form dimeric complexes with identical units and with different isoforms. As previously proposed, the diversity of acyl-CoA synthetase activities of the many ACSL6 enzyme species might represent an adaptation mechanism of an enucleated cell, the red blood cell, to the absolute necessity to repair damaged lipids and to maintain the lipid composition of the membrane [1,23].

The activation of fatty acids to acyl-CoA is required for many fatty acid utilizing pathways in physiology. All plasma membranes need to maintain a defined lipid composition, both in relative amount of the different classes and in molecular species within each class. Acylation of lysophospholipids with different acyl-CoA species is an essential step, and ACSL enzymes play a central role in this process [1]. In the red blood cell membrane, ACSL6 is responsible of maintaining the phospholipid composition of this enucleated cell lacking *de novo *lipid synthesis. For the iron-containing oxygen-carrying red blood cell, the activities of ACSL6 isoforms are especially important for the repair of phospholipids damaged by oxidation. ACSL6 is one of the major forms expressed in neuronal cells, and as in other cells that rapidly turn-over their plasma membrane lipids, activities of the different ACSL6 complexes might be essential to their plasma membrane plasticity [1]. An understanding of how the different isoforms of ACSL6 act, and interact, is essential to determine the mechanisms sustaining the formation of the various acyl-CoA species needed to maintain plasma membrane composition. Additional enzymatic studies combined with structural information are needed to better understand the function of these enzymes in physiologic processes.

## Methods

### DNA Manipulations

The cloning of cDNAs of human ACSL6 spliced variant 1, 2 and 3 into the expression vector pET28a (Novagen) with a hexahistidine tag at the N-terminus, has been previously published [8]. Removal of the amino-terminus of isoform 1 and 2 (residues 1 to 42) was performed by PCR with the set of primers Hs.ACSL6-130/fwd and Hs.ACSL6-end/rev (Table 1). The two amplicons were cloned at the *Nhe*I and *Hind*III restriction sites of pET28a. Compared to the full-length version, the ΔN-ACSL6_v1 (dubbed ΔN-Y-Gate) and ΔN-ACSL_v2 (dubbed ΔN-F-Gate) constructs lack the first 43 residues, which removed the trans-membrane spanning segment predicted at position +21 to 42 [1,8]. Removal of the motif carrying the Gate-domain (construct ΔGate) was performed by PCR. One fragment was amplified from nucleotides +1 to +915 (1^st ^half) with primers L6-v3-Nhe with L6-Gate/rev, and a second fragment from +993 to +2094 (2^nd ^half) with primers L6-Gate/fwd with Hs-ACSL6-end/rev. After PCR amplification and purification from agarose gel, the two amplicons were ligated to each other using a *Sal*I restriction site generated at the 3' end and 5' end of the 1^st ^and 2^nd ^fragment, respectively. Truncation of the N-terminus of the ΔGate construct to create the construct ΔN-ΔGate was performed as described above.

**Table 1:** List of oligonucleotides used in this study.

Name	sequence 5' to 3'
Hs.ACSL6-130/fwd	AGCTAGCTTCACTCACCGGCCAAAGGCC
Hs.ACSL6-end/rev	GGAAGCTTCACATGGAGATTGAGTAAAGCTCTTC
L6-v3-Nhe	ACTATAGCTAGCCAGACACAGGAGATCCTG
L6-Gate/rev	AAAGGGTCGACCTCTGTCACTTTCAGAAAGCC
L6-Gate/fwd	AAAGGGTCGACTCTGTCGTCTATTGCCACGGAG
Y319W-rev	ACATGTGTGCTAAAGGCAACCAGGAAATGTGCACATCCGCA
Y319A-rev	CATGTGTGCTAAAGGCAAAGCGGAAATGTGCACATCCGCA
Y319F-rev	ATGTGTGCTAAAGGCAAAAAGGAAATGTGCACATCCG
F319W	GATGTGCTCATCTCCTGGCTGCCTCTGGCTCACA
F319A	CGATGTGCTCATCTCCGCCCTGCCTCTGGCTCAC
F319Y-rev	ATGTGAGCCAGAGGCAGATAGGAGATGAGCACATCG
F/Y-L316H-rev	GGCAGATAGGAGATGTGCACATCGTCCTGTC
F-L316H-rev	GGCAGGAAGGAGATGTGCACATCGTCCTGTC
Y-H316L	CACTTGTGCGGATGTGCTCATTTCCTATTTGCCTT
Y/F-H316L	CACTTGTGCGGATGTGCTCATTTCCTTTTTGCCTT
Cola-BamH	ACTATAGGATCCGCAGACACAGGAGATCCTGAGG
Cola-Pml	ACTATACACGTGCAGACACAGGAGATCCTGAGG
Cola-Hind	CTATGCAAGCTTTCACATGGAGATTGAGTAAAGC
Cola-Xho	CTATGCCTCGAGCATGGAGATTGAGTAAAGCTC
ACSL6-EagI	TACTCGGCCGAGACACAGGAGATCCTGAGG
ΔN-EagI	TACTCGGCCGCTCACCGGCCAAAGGCCTTGC
HA-fwd	ATGTACCCATACGATGTTCCAGATTACGCTAGAT
HA-rev	ATCTAGCGTAATCTGGAACATCGTATGGGTACAT

Site-directed mutagenesis experiments were performed with the QuikChange Multi Site-directed Mutagenesis kit (Stratagene) according to the manufacturer instruction. Primers were designed with the QuikChange^® ^Primer Design Program (Stratagene). The presence of the intended nucleotide change(s) and the absence of unwarranted mutations were verified by full-length sequencing of the constructs.

Detection of protein-protein interactions was performed in *E. coli *using the BacterioMatch II Two-hybrid system (Stratagene). As indicated in Figure 9 and Additional file 2, the different isoforms were cloned by PCR at the *Not*I/*BamH*I restriction sites of the bait vector (pBT) and of the target vector (pTRG). Primers were designed to introduce an *Eag*I and a *Xho*I site at the 5' and 3' end, respectively, of the ACSL6 amplicons. Plasmids were propagated into the *E. coli *strain XL1-Blue MRF' Kan (Stratagene). All constructs were verified by sequencing and expression of the different fusion proteins was verified by immuno-detection with an affinity-purified anti-ACSL6 antibodies [8]. Results are presented in Additional file 2, panel C. Expression of the fused proteins was induced with a concentration of IPTG of 30 μM. Interaction analysis was performed in *E. coli *BacterioMatch II Validation reporter cells, carrying a *his*B mutation and the HIS3 reporter cassette, according to the manufacturer instructions (Stratagene).

Co-expression of isoforms fused at their amino-terminus with an hexahistidine-tag, or at their carboxy-terminus with a S-tag, were performed in *E. coli *cells transformed with various construct made in the pCOLA-DUET vector of Novagen. With this vector, two constructs can be cloned under the control of an identical promoter and expressed simultaneously upon addition of IPTG at a concentration of 100 μM in the culture medium. N-terminally His-tagged constructs were obtained by PCR cloning at the *BamH*I and *Hind*III restriction sites, whereas C-terminally S-tagged constructs were cloned at the *Nae*I and *Xho*I restriction sites. The two primer sets were designed to introduce *BamH*I and *Hind*III sites and *Pml*I and *Xho*I sites, respectively. The two primer sets were Cola-BamH with Cola-Hind, and Cola-Pml with Cola-Xho. Correct fusion of the different constructs was verified by sequencing, and expression of the fusion proteins was confirmed by immuno(affinity)-detection with an anti-Histidine antibody (Pierce) and S-protein (Novagen), respectively (see below).

### Expression and immunodetection of ACSL6

ACSL6 isoforms cloned into pET28a and pCOLA-DUET vectors were transformed into *E. coli *BL21(DE3) cells (Novagen). Expression and detection with an HRP-conjugated anti-Histidine or anti-ACSL6 antibody were performed as previously described [8]. Cells were disrupted in breakage buffer containing Tris-HCl 50 mM, pH 8.0, EDTA 5 mM, NaCl 0.3 M, DTT 5 mM and PMSF 0.1 mM, by two passages in a French press cell at 12,000 psi. for The lysate was cleared by centrifugation at 8,000 g for 20 min at 4°C. To prepare the membrane and soluble fractions, the total extract was subjected to centrifugation at 100,000 g for 60 min at 10°C. The supernatant represented the soluble fraction. The pellet represents the membrane fraction and was suspended in breakage buffer supplemented with 10% glycerol and, stored frozen at -80°C. Detection of the S-tagged constructs with a HRP-conjugated-(S-protein) (Novagen) was performed under the same condition used with the anti-His antibody. Expression of isoforms cloned in the pBait and pTRG vectors was performed in M9 salt synthetic medium with 0.2% glucose supplemented with 1 mM MgS0_4_, 1 mM thiamine-HCl, 0.2 mM adenine, with 30 μM IPTG (induced condition), and with 5 mM 3-amino-1,2,4-triazole, freshly made in DMSO at 1 M, and when appropriate, supplemented with 25 μg/ml kanamycin, 25 μg/ml chloramphenicol, and 10 μg/ml tetracycline, according to the manufacturer instructions (Stratagene). Cells were grown at 37°C for 24 to 48 hrs or at 30°C for 48 to 72 hrs. Production of the different fusion proteins was analyzed by immuno-detection of protein extracts of cells grown in presence of IPTG with an affinity-purified anti-ACSL6 antibody which recognized all the isoforms and the ΔN constructs fused to either the lambda-cI protein (Bait vector set) or the amino-terminal domain of the RNA polymerase alpha subunit (Additional file 2). The analysis was done with cells transformed with only one of each of the constructs so as to confirm that each construct was correctly fused and produced at level comparable to each other. Predicted molecular mass (in kDa) of the fusions were: cI-isoform 1, 103; cI-isoform 2, 103; cI-isoform 3, 95; cI-ΔN-isoform2, 99; RNAPa-isoform 1, 105; RNAPa-isoform 2, 105; RNAPa-isoform 3, 97.

### Acyl-CoA synthetase assay

All measurements were performed with membrane fractions obtained from *E. coli *BL21(DE3) cells expressing the different isoforms and constructs. Prior to measurements, control experiments were performed with the different enzyme preparations to determine the correct amount of proteins that will not process more than 20% of the radio-labeled fatty acids under the reaction condition and the period of the incubation. The particular reaction conditions of each assay are given in the legend of the figures. In most experiments, 0.25 to 10 μg of proteins were used and the reactions were performed at 30°C for an incubation period ranging from 0 to 8 min. Unless indicated otherwise, reactions were performed in a final volume of 150 μl and initiated by the addition of 130 μl of a mixture containing the enzyme to the fatty acid dissolved in 20 μl. The fatty acid was usually first dispatched in glass tubes and pre-warmed at 30°C before addition of the pre-warmed enzyme mixture. When appropriate, protein diluted in 10 μl was first dispatched in the tubes and reactions were initiated by addition of 140 μl of the reaction mixture containing the fatty acids mixture. ^14^C-labeled fatty acid suspensions were usually provided at a concentration of 4 μM (unless otherwise indicated in legend of figures) with a specific activity of 50-54 mCi/mmol. Dried fatty acids were suspended in 20 μl of 13.5 mM NaHCO_3 _and 0.55 mM Triton X-100 and were diluted to 1.8 mM NaHCO_3 _and 74 μM Triton X-100 in the final reaction volume. The 130 μl enzyme mixture was diluted with the fatty acid buffer to a final concentration of 0.1 M Tris.HCl pH 8.0, 1 mM DTT, 20 mM MgCl_2_, 20 mM ATP and 0.5 mM CoA. The reaction was stopped by addition of 2.25 ml of isopropanol/heptane/2 M H_2_SO_4 _(40:10:1, v/v) and vigorous vortexing. Newly synthesized ^14^C-acyl-CoA was separated from un-esterified ^14^C-fatty acid by successive addition of 1 ml of heptane and 1 ml of H_2_O, and vigorous vortexing. The two phases were separated by centrifugation at 500 g for 3 min. The upper organic phase was carefully removed and collected in a scintillation vial. The lower aqueous phase was then extracted twice with 2 ml heptane saturated with 4 mg/ml palmitic acid, and once with 2 ml heptane. Each of the upper phases were collected and pooled with the first one. The amounts of newly synthesized ^14^C-acyl-CoA and of[^14^C-fatty acid were measured by scintillation counting in the lower and upper phases, respectively. For each reaction, the total amount of ^14^C present was calculated by addition of the dpm values of the upper and lower phases. Control reactions were performed with membranes obtained from cells carrying the vector pET28a and the dpm values obtained were subtracted to those obtained with the enzyme preparations. For calculation of the kinetic parameters, data were displayed as a scatter plot of activity rates in function of the concentration of substrate (Figure 2) using Prism graphing software version 5 (GraphPad Software, Inc.), and the kinetic values were obtained by non-linear regression of the data computed by the software.

Thin-layer chromatography detection of the acyl-species was performed as described in [24]. Reactions were performed in 15 μl with 10 μM ^14^C-oleic acid and 1 μg of membrane proteins. After 10 min incubation at 30°C, the reaction was stopped with 1.6 μl of acetic acid 50% and spotted on a silica plate. Along with standards, species were separated at 4°C for 4-5 hrs in a solvent system made of 1-butanol/acetic acid glacial/water (80/25/40; v/v). The plates were dried and analyzed with a PhosphoImager Storm Scanner (Molecular Dynamics, GE Healthcare).

## Authors' contributions

ES conceived the study and its design. ES, NPD and MS carried out the experiments. FK participated in the conception of the study and interpretation of data. ES and FK wrote the manuscript. All authors read and approved the manuscript.

## Supplementary Material

Additional file 1 **Immuno-detection of full-length and mutant ACSL6 proteins**. Full-length ACSL6 isoform 2 (F-Gate) and ΔN-truncated version of isoform 1 [ΔN-(Y-Gate)] and, their respective mutants obtained by side-directed mutagenesis of the H/L residue pair at position 316 and of the F/Y residue pair at position 319 (see the result section), were expressed in *E. coli *with an hexahistidine tag at their N-terminus. Membrane fractions were obtained as described in the methods section. Proteins (10 μg) were separated on denaturing SDS-PAGE 7.5% gel and stained with an HRP conjugated anti-histidine antibody (India-His, Pierce). The molecular weight standard (Dual Precision Plus protein, Bio-Rad) is indicated on the left. Position of the monomer and dimer species is indicated by arrow on the right. Some extra bands, that may represent partially denatured oligomeric complex and aggregates (bands between the dimer and monomer bands) and unfinished translated products (detected by mean of the hexahistidine tag present at their N-terminus) as well as degradation products, can be seen and are indicated with asterisk. Click here for file

Additional file 2 **Two-hybrid interaction analysis of oligomer formation**. Isoform 1, 2 and 3 were cloned in Bait and Target vectors. The truncated ΔN form of isoform 2 was cloned in the Bait vector. Including the empty vectors, all the different plasmid combinations were transformed in the *hisB*-derivatived *E. coli *strain carrying the reporter HIS3 cassette. **A**. Growth tests were performed on solid media in absence (not induced) or presence (induced) of 30 μM IPTG and without or with addition of 5 mM 3'-AT (drug). E. coli strain transformed with the two vectors (-/-) grew in absence but not in presence of the drug; positive control (+/+; Gal4/Gal11) grew in presence of the drug and IPTG. Plates on the left side represent cells transformed with the 4 Bait constructs, as indicated on the top of each columns, and with the 3 Target constructs, as indicated on the left side. Note that some combinations resulted in poor growth. **B**. Growth tests were performed in liquid media in presence of the 30 μM IPTG without or with 5 mM 3'-AT, at 37°C. The histogram represent the ratio of the growth yield values of the cultures (OD_600nm_) with and without 3'-AT obtained with each of the different combination of constructs (as seen on panel A). Bait and target constructs combinations are indicated on the left of each column of the histogram. **C**. Production of the different fusion proteins was analyzed by immuno-detection, as described in the Methods section. Click here for file
