# Supplementary figures and images for: Activity of the acyl-CoA synthetase ACSL6 isoforms: role of the fatty acid Gate-domains
Source: BMC Biochem. 2010 Apr 29;11:18. doi: 10.1186/1471-2091-11-18 (PMC2868784; doi:10.1186/1471-2091-11-18)

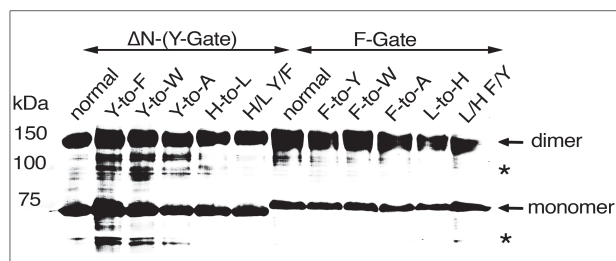

Supplement: Additional file 1 — Immuno-detection of full-length and mutant ACSL6 proteins. Full-length ACSL6 isoform 2 (F-Gate) and ΔN-truncated version of isoform 1 [ΔN-(Y-Gate)] and, their respective mutants obtained by side-directed mutagenesis of the H/L residue pair at position 316 and of the F/Y residue pair at position 319 (see the result section), were expressed in E. coli with an hexahistidine tag at their N-terminus. Membrane fractions were obtained as described in the methods section. Proteins (10 μg) were separated on denaturing SDS-PAGE 7.5% gel and stained with an HRP conjugated anti-histidine antibody (India-His, Pierce). The molecular weight standard (Dual Precision Plus protein, Bio-Rad) is indicated on the left. Position of the monomer and dimer species is indicated by arrow on the right. Some extra bands, that may represent partially denatured oligomeric complex and aggregates (bands between the dimer and monomer bands) and unfinished translated products (detected by mean of the hexahistidine tag present at their N-terminus) as well as degradation products, can be seen and are indicated with asterisk. [file 1471-2091-11-18-S1.PDF]

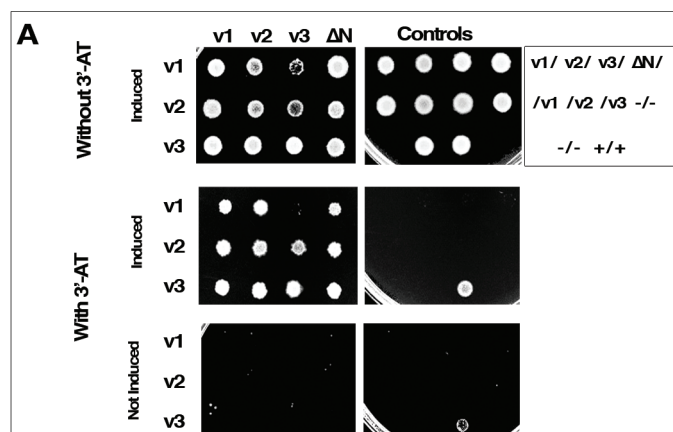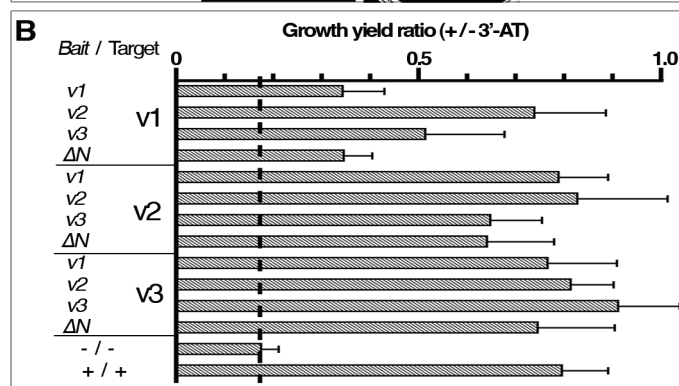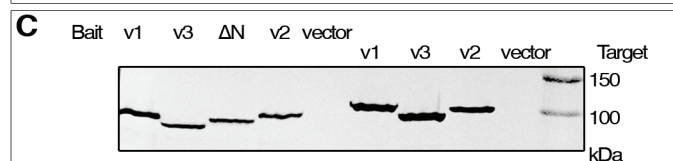

Supplement: Additional file 2 — Two-hybrid interaction analysis of oligomer formation. Isoform 1, 2 and 3 were cloned in Bait and Target vectors. The truncated ΔN form of isoform 2 was cloned in the Bait vector. Including the empty vectors, all the different plasmid combinations were transformed in the hisB-derivatived E. coli strain carrying the reporter HIS3 cassette. A. Growth tests were performed on solid media in absence (not induced) or presence (induced) of 30 μM IPTG and without or with addition of 5 mM 3'-AT (drug). E. coli strain transformed with the two vectors (-/-) grew in absence but not in presence of the drug; positive control (+/+; Gal4/Gal11) grew in presence of the drug and IPTG. Plates on the left side represent cells transformed with the 4 Bait constructs, as indicated on the top of each columns, and with the 3 Target constructs, as indicated on the left side. Note that some combinations resulted in poor growth. B. Growth tests were performed in liquid media in presence of the 30 μM IPTG without or with 5 mM 3'-AT, at 37°C. The histogram represent the ratio of the growth yield values of the cultures (OD600nm) with and without 3'-AT obtained with each of the different combination of constructs (as seen on panel A). Bait and target constructs combinations are indicated on the left of each column of the histogram. C. Production of the different fusion proteins was analyzed by immuno-detection, as described in the Methods section. [file 1471-2091-11-18-S2.PDF]
